# An emerging link between lncRNAs and cancer sex dimorphism

**DOI:** 10.1007/s00439-023-02620-7

**Published:** 2023-12-14

**Authors:** Ikrame Naciri, Maria D. Andrade-Ludena, Ying Yang, Mei Kong, Sha Sun

**Affiliations:** 1 Department of Developmental and Cell Biology, School of Biological Sciences, University of California Irvine, Irvine, CA 92697 USA; 2 Department of Molecular Biology and Biochemistry, School of Biological Sciences, University of California Irvine, Irvine, CA 92697 USA

## Abstract

The prevalence and progression of cancer differ in males and females, and thus, sexual dimorphism in tumor development directly impacts clinical research and medicine. Long non-coding RNAs (lncRNAs) are increasingly recognized as important players in gene expression and various cellular processes, including cancer development and progression. In recent years, lncRNAs have been implicated in the differences observed in cancer incidence, progression, and treatment responses between men and women. Here, we present a brief overview of the current knowledge regarding the role of lncRNAs in cancer sex dimorphism, focusing on how they affect epigenetic processes in male and female mammalian cells. We discuss the potential mechanisms by which lncRNAs may contribute to sex differences in cancer, including transcriptional control of sex chromosomes, hormonal signaling pathways, and immune responses. We also propose strategies for studying lncRNA functions in cancer sex dimorphism. Furthermore, we emphasize the importance of considering sex as a biological variable in cancer research and the need to investigate the role lncRNAs play in mediating these sex differences. In summary, we highlight the emerging link between lncRNAs and cancer sex dimorphism and their potential as therapeutic targets.

## Introduction

The incidence of cancer, therapeutic response, and overall survival of cancer patients differ between men and women (Henley et al. 2020; Islami et al. 2021). Males are generally more likely to develop cancer. The National Cancer Institute reported through the Surveillance Epidemiology and End Results (SEER) database that for all cancers combined in the United States from 2016 to 2020, the age-adjusted incidence rate (and 95% confidence interval) per 100,000 was 477.8 ± 0.7 for males and 412.8 ± 0.6 for females. The same data analysis for the cancer mortality rate was 177.5 ± 0.3 for males and 128.7 ± 0.2 for females. The sex disparity in cancer is not restricted to any country or region. For the year 2020, the Global Cancer Observatory GLOBOCAN database provided statistics for 36 cancer types across 185 countries, with an age-adjusted incidence rate of 222.0 for males and 186.0 for females. In 2020, the world’s cancer mortality rate was 120.8 for males and 84.2 for females. We present in Table 1 the incidence rates for major cancers based on sex. Males have higher incidence rates for many types of cancer.

**Table 1:** Comparison of sex-specific incidence rates (per 100,000) for selected cancers with a male-to-female ratio indicating male bias (in blue) versus female bias (in pink)

Cancer type	SEER 2020 (USA)^a^			Globocan 2020 (worldwide)^b^		
	Male	Female	Ratio	Male	Female	Ratio
Lung	48.8	39.4	1.2	31.5	14.6	2.2
Colorectum	37.7	28.8	1.3	23.4	16.2	1.4
Melanoma	23.4	14.9	1.6	3.8	3	1.3
Non-Hodgkin lymphoma	20.8	14.2	1.5	6.9	4.8	1.4
Bladder	29.2	7	4.2	9.5	2.4	4
Kidney	21.9	10.6	2.1	6.1	3.2	1.9
Thyroid	6.6	17	0.4	3.1	10.1	0.3
Leukemia	16.1	10.1	1.6	6.3	4.5	1.4
Pancreas	14.6	11.4	1.3	5.7	4.1	1.4
Oral cavity	16.1	6.1	2.6	6	2.3	2.6
Liver	12.6	4.9	2.6	14.1	5.2	2.7
Myeloma	7.9	5.3	1.5	2.2	1.5	1.5
Stomach	8.2	4.7	1.7	15.8	7	2.2
Brain, nervous system	7.1	5	1.4	3.9	3	1.3
Larynx	4.1	0.9	4.6	3.6	0.45	8
Hodgkin lymphoma	2.7	2.2	1.2	1.2	0.8	1.5
Gallbladder	0.9	1.4	0.6	0.89	1.4	0.6
Mesothelioma	1.1	0.4	2.8	0.46	0.17	2.7
Kaposi sarcoma	0.7	0.1	7	0.54	0.25	2.2
Breast	–	118.8	–	–	47.8	–
Prostate	104.1	–	–	30.7	–	–
Testis	5.9	–	–	1.8	–	–
Ovary	–	9.3	–	–	6.6	–
Cervix Uteri	–	6.9	–	–	13.3	–
Vulva	–	2.3	–	–	0.85	–

For all listed cancer types in SEER 2020 and Globocan 2020 data, the differences in incidence rates between males and females are statistically significant with* P* < 0.05

^a^SEER 2020 USA data from the SEER Incidence Data at https://seer.cancer.gov/statistics-network/explorer

November 2022 Submission (1975–2020), SEER 22 registries https://seer.cancer.gov/registries/terms.html

^b^Globocan 2020 (worldwide) data from the Global Cancer Observatory GLOBOCAN database at https://gco.iarc.fr/

The numbers reported in Table 1 include sex-specific reproductive cancer types, such as ovarian and prostate cancers. The common non-reproductive cancers include the types that have high male-to-female ratios: colorectal cancers, lung and liver, and non-Hodgkin lymphoma. The analysis of SEER 2020 (USA) identified Kaposi sarcoma as having the highest male-to-female incidence rate ratio. In addition to breast cancer, which is rare in males, only a few cancers are more common in females, which is similar to what was previously noted (Dorak and Karpuzoglu 2012). In the case of gallbladder and thyroid cancer types, the male-to-female incidence rate ratio is less than 1.0. Although lifestyles are known to contribute to these differences, genetics also play an important role, and the molecular mechanisms involved are largely unknown. Despite traditional beliefs that sex hormones and hormonal regulation are the main explanatory factors for sexual dimorphism in cancer, accumulating evidence suggests that additional genetic and epigenetic mechanisms are at play. However, the specific molecular and cellular mechanisms responsible for sexual dimorphisms in cancer incidence and therapeutic responses are still in the early stages of discovery (Sandovici et al. 2022).

Non-coding RNAs (ncRNAs; RNAs that do not encode proteins) play critical roles in gene regulation (Mattick and Makunin 2006; Nair et al. 2020). Long noncoding RNAs (lncRNAs) are larger than 200 nucleotides and are important building blocks of gene regulatory networks in all eukaryotes (Kopp and Mendell 2018; Rinn and Chang 2012; Yao et al. 2019). LncRNAs share many similarities to messenger RNA (mRNA), including transcription by RNA Polymerase II, and undergoing co- and post-transcriptional processing events such as splicing, 5′ capping, and 3′ polyadenylation (Cuykendall et al. 2017; Mattick et al. 2023; Quinn and Chang 2016). The human genome encodes thousands of lncRNAs, which represent potentially key sources of gene regulatory adaptation (Djebali et al. 2012). Analyzing lncRNAs is challenging as they are often expressed at low levels. Some investigators consider lncRNAs to be transcriptional “noise”. Therefore, the functions for most lncRNAs remain obscure and their biological importance is disputed. With advances in whole-genome technologies, increasing research on lncRNAs has highlighted their important role in a wide range of cellular processes. LncRNAs participate in chromatin remodeling and transcription, splicing, translation, and processing, localizing, and stabilizing other RNAs (Mattick et al. 2023).

An important role for lncRNAs in tumor initiation and progression has been established in recent years (Bhan et al. 2017; Jiang et al. 2019). Their tissue-specific expression makes them attractive for diagnostic and therapeutic purposes. For instance, the lncRNA *PCA3* (Prostate Cancer Antigen 3) is used as a diagnostic marker for prostate cancer, and it can be easily found in urine samples (Taheri et al. 2022); the lncRNA *HOTAIR* (HOX antisense intergenic RNA) is involved in hormone therapies resistant in breast cancer (Xue et al. 2016). Sex-biased expression of lncRNAs plays a role in modulating the regulatory pathways underlying sexual dimorphism in brain and cardiovascular disorders (Hartman et al. 2018; Issler et al. 2020; Jusic et al. 2020; Reinius et al. 2010; Simchovitz-Gesher and Soreq 2020). Cancer sex dimorphism may also be influenced by lncRNA expression.

**Fig. 1:**
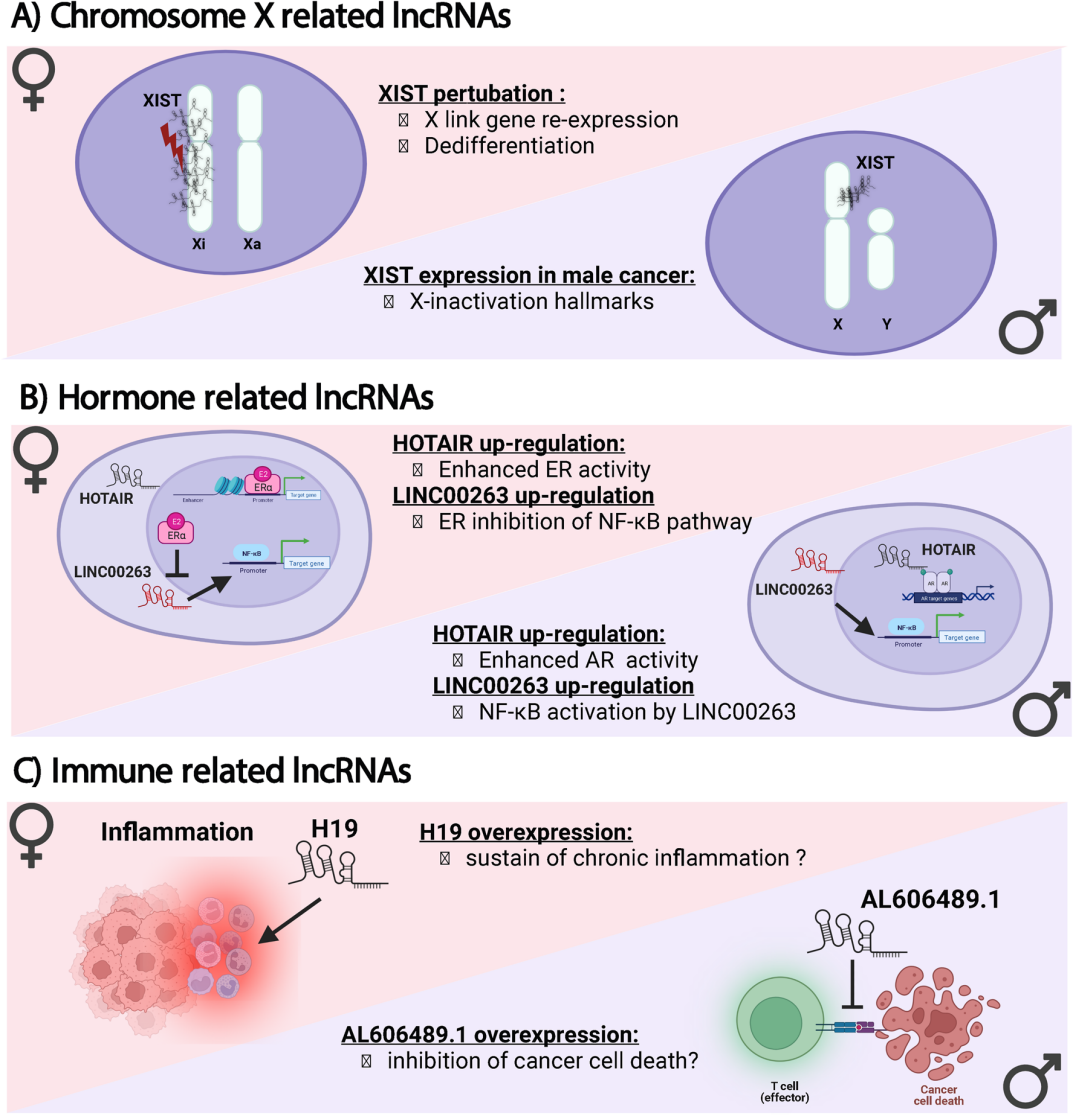
LncRNA in cancer sex dimorphism. **A** X-chromosome-related lncRNA: *XIST* lncRNA is involved in reproductive cancer like breast and cervical cancer, but also in blood cancer. Its disruption led to the reactivation of X-linked genes and cell dedifferentiation. *XIST* is active in non-reproductive cancers in males, leading to X chromosome inactivation hallmarks such as DNA methylation. **B** Hormones-related lncRNA: Example of lncRNAs that interacts with hormonal pathways and contributes to the progression of cancer. *HOTAIR* lncRNA is involved in both the estrogen receptor (ER) and androgen receptor (AR) transcriptional programs, resulting in different outcomes in reproductive cancer. *LINC000263* lncRNA activates the NF-κB pathway in male lung cancer patients. The same pathway is inhibited in female lung cancer patients due to the estrogen receptor competition with the NF-κB transcription factor. **C** Immune-related lncRNAs: Example of lncRNAs that plays a role in cancer progression through immune system dysregulation. While the functions of *H19* and *AL606489.1* in cancer progression remain unclear, they could play roles in pro-tumorigenic inflammation and in inhibiting immunotherapies-induced cell death, respectively. Created with BioRender.com

In this review, a number of potential mechanisms by which lncRNAs may influence sexual dimorphism in cancer are discussed, including transcriptional control of sex chromosomes, hormone signal transduction pathways, and immune responses (Fig. 1). We address sexual dimorphism as differences caused or driven by sex. A sexual dimorphism is characterized by dichotomous features, such as ovaries and testes (Rubin et al. 2020). While we describe sex differences in cancer incidence and mortality, such differences may be attributed to sexual dimorphism. In this context, we will also highlight the approaches that can be used to identify cancer-sex-specific lncRNAs. The discussion focuses on lncRNAs that play a role in female-reproductive cancers such as breast and ovarian, male-reproductive cancers such as testicular and prostate, as well as non-reproductive cancers such as liver, colorectal, lung, brain, skin, blood, and thyroid.

## X-chromosome lncRNAs and cancer sex dimorphism

Although cancer is generally considered a genetic disease, phenotypic plasticity and epigenetic reprogramming have been recognized as emerging hallmarks and enabling characteristics necessary for tumor growth and progression (Hanahan 2022; Hanahan and Weinberg 2011). From fertilization and through embryo development and adulthood, sex chromosomes establish major physiological differences between males and females. Male-specific transcriptional regulation in mammals begins with the Y chromosome and the Sex-determining Region Y (*SRY*) gene. Recently, *KDM5D* has been identified as a new Y-linked gene involved in sex differences in colon cancer and bladder cancer (Abdel-Hafiz et al. 2023; Li et al. 2023a). An overexpression of *KDM5D*, a histone demethylase, by the transcription factor *STAT3* results in epigenetic regulatory changes, leading to increased colon cancer metastasis in a mouse model (Li et al. 2023a). In bladder cancer, loss of Y chromosome (LOY) enhances tumor sensitivity to immunotherapies (Abdel-Hafiz et al. 2023). Nevertheless, the genetic difference between XX females and XY males resides largely in the X chromosome, which contains over 1000 genes important for cell survival and behavior. Males and females have different numbers of X-chromosomes, which could result in an imbalance in the dose of gene products associated with X during embryonic development. As a way to resolve this problem, one of the two X-chromosomes is transcriptionally silenced in female mammals through X-inactivation (Lyon 1961; Payer and Lee 2008; Wutz 2011). Furthermore, X-inactivation allows humans to tolerate sex chromosomes with abnormal numbers, such as 45, XO (Turner syndrome), 47, XXY (Klinefelter syndrome), or 47, XXX (Triple X syndrome) karyotypes. Early development in mammals depends on an effective dosage balance and failure would result in embryonic death. When X-inactivation is skewed or incomplete, developmental defects can be caused by dysregulation of X-linked genes. This results in diseases such as fragile X syndrome, Duchenne muscular dystrophy, and sex bias in systemic lupus erythematosus (Kirchgessner et al. 1995; Pyfrom et al. 2021; Syrett et al. 2019; Viggiano et al. 2016; Yu et al. 2021). Age-acquired skewed X-inactivation has recently been linked to cancer incidence (Roberts et al. 2022).

X-inactivation is an epigenetic hallmark of mammalian development (Fang et al. 2019; Payer et al. 2011). Two X-chromosomes in a female cell activate the expression of the X-Inactivation-Specific Transcript (*XIST*). In females, *XIST* is an lncRNA of 17-kb long that causes transcriptional inactivation of one of the two X-chromosomes, and this effect persists in all somatic cells throughout their life. LncRNA *XIST* and its flanking regulatory lncRNAs genes such as *JPX* (Just Proximal of Xist) and *FTX* (Five Prime to Xist) control the initiation of X-inactivation and the choice of which one of the two X-chromosomes to silence (Furlan et al. 2018; Karner et al. 2020; Rosspopoff et al. 2023; Sun et al. 2013). For both humans and mice, the lncRNA *XIST*/*Xist* regulates dosage compensation by randomly inactivating one of the X-chromosomes during post-implantation development in the female. Based on extensive studies in mice, *Xist* is transcribed from the X chromosome to be inactivated; the *Xist* lncRNA coats the X chromosome and recruits chromatin protein complexes to spread in *cis*, resulting in altered chromatin modifications and transcriptional silencing along the X chromosome (Brockdorff et al. 2020; Jacobson et al. 2022; Li et al. 2022; Patrat et al. 2020).

A link between *XIST*, X-linked gene expression, and cancer has been demonstrated in mice and humans (Richart et al. 2022; Xing et al. 2018; Yildirim et al. 2013). Female mice with *Xist*-deletion develop marrow fibrosis, leukemia, and histiocytic sarcoma in female mice; male mice with the same deletion have no defects (Yildirim et al. 2013). In humans, *XIST* is dysregulated in breast cancer and loss of *XIST* is a common feature of breast tumors that have a poor prognosis (Richart et al. 2022; Xing et al. 2018). It is believed that *XIST* loss triggers X chromosome reactivation, resulting in the overexpression of X-linked genes that contributes to cancer development (Chaligné et al. 2015). It has been found that, however, *XIST* loss does not cause a massive X chromosome reactivation in human mammary stem cells. Only a few genes, including the chromatin mediator *MED14* (mediator of RNA polymerase II transcription subunit 14), are reactivated. The overexpression of *MED14* impairs the differentiation and homeostasis of mammary stem cells (Richart et al. 2022). Additionally, the study found that loss of *XIST* in mammary stem cells results in tumorigenesis in mice carrying an oncogenic mutation. The loss of *XIST* alone does not lead to cancer progression in stem cells; however, multiple hits are necessary to promote cancer development. To clarify the exact role of *XIST* in cancer development, additional examples and evidence will be needed.

A hallmark of cancer is the maintenance of cell identity and plasticity (Hanahan 2022). Loss of *XIST* can give cancer stem cells the advantage of maintaining their pool of stem-like cancer cells and giving them a proliferative advantage in a cancer context, in which cancer stem cells are defined as subpopulations of cancer cells capable of renewal and differentiation. A loss of *XIST* in an ovarian cancer cell line leads to an increase in cancer stem cells, as indicated by the expression of cancer stem cell markers such as *OCT4* and *SOX2* (Huang et al. 2020). These studies highlight the importance of *XIST* lncRNA function in maintaining the transcriptional status of the X chromosome and preserving the cell identity and plasticity. While X-inactivation appears to confer protection from cancer in females and may contribute to the male bias in general cancer types, it is important to determine the expression status of *XIST* in female-reproductive cancers such as breast and ovarian cancer. However, *XIST* can be somatically activated in a subset of male human cancers, even though X-inactivation and *XIST* expression are generally absent from normal male tissues. Some of these cancers exhibit X- inactivation-like characteristics, such as higher levels of DNA methylation on chromosome X and silenced expression of X-linked genes (Sadagopan et al. 2022). Thus, understanding the molecular mechanisms behind the function of *XIST* lncRNA in cancer progression will improve diagnosis and treatment for both males and females.

## Hormone-interacting lncRNAs and cancer sex dimorphism

Sex hormones play a significant role in the initiation and progression of cancer in both males and females. Hormones play a key role in reproductive-specific cancers, such as breast and ovarian cancers in females and prostate cancers in males. The inherent dimorphic nature of the reproductive organs arises from distinct mechanisms unique to each sex, which are regulated by the endocrine gland (Chou and Henderson 2014; Henderson 2005). Beyond this, hormones, such as estrogen and androgens, have a substantial impact on the development and prognosis of cancers in sexually different ways. For instance, estrogen, a critical sex hormone in both male and female systems, displays contrasting effects in female- and male-specific cancers. While estrogen decreases tumor formation in cancers primarily affecting men, such as liver and colon cancer, it paradoxically increases tumor formation and progression in cancers primarily affecting women, such as meningiomas and thyroid cancer (Rubin et al. 2020). In breast cancer development, estrogen signaling plays a crucial role, but the underlying mechanisms are not yet fully known (Clusan et al. 2023).

The lncRNA Homeobox transcript antisense intergenic RNA (*HOTAIR*) is implicated in estrogen and androgen receptor signaling. A specific function of *HOTAIR* is found to upregulate nuclear estrogen receptors and affect estrogen-related gene expression (Xue et al. 2016). By also interacting with chromatin, *HOTAIR* is involved in breast cancer migration and promotes tumor metastasis via altering chromatin status (Gupta et al. 2010). The lncRNA *HOTAIR*, when overexpressed, can upregulate estrogen, which can contribute to cervical cancer progression and prognosis, and is elevated in both estrogen receptor-positive (ER+) and triple-negative (TN) breast cancer through distinct mechanisms. In ER+ breast cancer, which accounts for 70–80% of human breast cancer tumors (Sohail et al. 2020), *HOTAIR* is induced through estradiol (E2), the main form of estrogen, by binding to estrogen response elements (EREs) on its promoter region. This enhances recruitment of RNA polymerase II by increasing histone acetylation and histone H3 lysine-4 trimethylation (Bhan et al. 2014). 20% of breast cancer cases are TN breast cancer (Yager and Davidson 2006). Here, estrogen stimulates *HOTAIR* via the G protein-coupled receptor and suppresses miRNA miR-148a, which negatively affects *HOTAIR* levels (Tao et al. 2015).

In cancers specific to males, lncRNAs play in conjunction with hormones related to the male system such as androgenic hormones. Androgenic hormones, testosterone being a prominent example, can exert effects through lncRNA regulation mechanisms. For instance, the lncRNA Androgen Receptor Regulated Long Non-coding RNA 1 (*ARLNC1*) regulates androgen receptor signaling and is one of the most differentially expressed androgen receptor-regulated genes in prostate cancer. *ARLNC1* functions by binding to the androgen receptor, promoting its stability and enhancing transcriptional activity through a positive-feedback loop. As a result, *ARLNC1* promotes androgen receptor-dependent prostate cancer cell proliferation (Zhang et al. 2018).

The effects of sex hormones and their interacting lncRNAs can also be sexually dimorphic on non-reproductive cancers. Recent studies have shown that lncRNAs that interact with estrogen receptors, such as Metastasis-Associated Lung Adenocarcinoma Transcript 1 (*MALAT1*), and lncRNA-H19 (*H19*), are enhanced by estrogen receptors in lung and thyroid cancers. In lung cancer, *MALAT1* knockdowns alternatively splice the 5′ untranslated region of *ESR1*, the gene that codes for estrogen receptor α (Arun et al. 2016). In the case of *H19*, estradiol (E2) promotes *H19* transcription through estrogen receptor β, which induces stem-like properties in thyroid cancer (Li et al. 2018). Both lncRNAs are overexpressed by estrogen receptors, and in the case of *MALAT1*, it causes worse survival outcomes in female patients with estrogen receptor β-positive lung cancer than those in estrogen receptor β-negative lung cancer (Yu et al. 2019). In melanoma, lncRNAs that bind to the androgen receptor and regulate the transcription of growth-regulatory genes, such as SRA-like Non-coding RNA (*SLNCR*), contribute to poorer prognosis for men compared to women. *SLNCR* binds with the androgen receptor adjacent to *SLNCR*’s conserved region, and overexpression leads to increased melanoma invasion (Schmidt et al. 2019). In renal cancer, the lncRNA Suppressing Androgen Receptor in Renal Cell Carcinoma (*SARCC*) suppresses tumor growth by inhibiting androgen receptor functions, specifically through stabilizing the androgen receptor protein in males, leading to a repression of miR-142-3p. When overexpressed, *SARCC* inhibits downstream signals in the AKT, MMP, K-RAS, and P-ERK pathways (Zhai et al. 2017). It has been reported that the androgen receptor could induce renal cell carcinoma initiation, progression, and invasion, which may explain why men are more likely to develop renal cancer than women (Chen et al. 2015; He et al. 2014). Again, the lncRNA *HOTAIR* plays a role in estrogen and androgen receptor signaling and is regulated by estrogen receptor β in renal cell carcinoma. This regulation promotes tumor growth and invasion in both males and females. *HOTAIR* is also involved in regulating proliferating renal cell carcinomas and can control the transcription of androgen receptor targets, contributing to a more resistant response to antiandrogens in prostate cancer (Ding et al. 2018; Kumar et al. 2021).

The lncRNA *LINC00263* is overexpressed in several types of cancer. A higher expression is associated with poor prognosis in lung, renal cell carcinoma, colorectal cancer, and hepatic carcinoma, but is favorable in ovarian cancer, and has no significant effect in prostate and breast cancer. When comparing sex-specific differences in lung adenocarcinoma, colorectal cancer, or renal cell carcinoma patients, the expression of *LINC00263* was found to be higher in males than females. The authors pointed out a strong negative correlation with *XIST* as well as with estrogen receptor 1 expression, which may be an explanation for the difference in the expression of *LINC00263* between sexes. Additionally, estrogen can inhibit the activation of NF-κB signaling and reduce the activity of protein p65 within this pathway. Since *LINC00263* is expressed less in female patients than in male patients, especially in ER-negative breast cancer, an interaction between estrogen receptors may be inhibiting *LINC00263* expression. Hence, estrogen-induced inhibition of *LINC00263* might contribute to sex-specific differences in cancer progression (Liu et al. 2020).

## Sex-specific immune-related lncRNAs in cancer

One of the hallmarks of cancer is its ability to evade immune destruction, although the immune system can also promote tumorigenesis by creating an environment that is pro-inflammatory (Hanahan 2022). It is generally observed that women exhibit better immune responses than men, even when they are battling cancer (Klein and Flanagan 2016). Recent studies analyzing RNA-sequencing from solid tumors have revealed that women's tumor microenvironments contain more innate and adaptive immune cells, resulting in sex differences regarding molecular mechanisms for cancer cell immune invasion (Castro et al. 2020; Conforti et al. 2021). It is also well known that women respond better than men to cancer immunotherapies, such as anti-PD-1/anti-PD-L1 treatment. However, the molecular mechanisms behind sexual dimorphism in the immune response remain unclear (Conforti et al. 2021; Schafer et al. 2022). Therefore, there is an increasing interest in identifying sex-specific immune-related lncRNAs in cancer.

In females with hepatocellular carcinoma, the lncRNA *H19* is upregulated compared to male patients (Zhang et al. 2015). *H19* is an imprinted gene expressed by the maternal allele. Its role in tumorigenesis is unclear and is largely dependent on the type of cancer. *H19* is involved in a variety of cancer hallmarks, including the pro-oncogenic inflammatory environment that characterizes liver cancer (Tietze and Kessler 2020). Interestingly, it is also involved in liver fibrosis, an inflammatory condition in which innate and immune cells play a key role (Pellicoro et al. 2014). In addition, inflammation may facilitate tumor growth in some contexts. It has been suggested that *H19* in female hepatocellular carcinoma could cause a sustained inflammatory environment and promote tumor progression (Hiam-Galvez et al. 2021). The validation of this hypothesis will help us understand how sex-differential expression of *H19* affects liver cancer outcomes.

Another example is the lncRNA *AL606489.1*, which is differentially expressed between male and female lung cancer patients, with a higher level of expression in males. Furthermore, the expression of *AL606489.1* was negatively correlated with survival in male cancer patients only (Liang et al. 2022). The *AL606489.1* lncRNA is considered as an immune-related lncRNA in lung cancer and positively correlates with immune-related genes. In combination with four other lncRNAs (*AC068338.3*, *AL691432.2*, *TMPO-AS1,* and *AP000695*), it can provide prognosis for lung cancer patients based on an immune-related risk score incorporating the expression levels of these immune-related lncRNAs; a higher level of *AL606489.1* expression is associated with a higher risk score and increased mortality rate (Lu et al. 2021). There is also evidence that *AL606489.1* plays a role in ferroptosis, a type of programmed cell death dependent on intracellular iron (Guo et al. 2021). Immunotherapy-dependent ferroptosis relies on the ability of ferroptosis induction to enhance immune cell activity, allowing cancer cells to be more sensitive to immunotherapies (Gong et al. 2022). It is unclear how *AL606489.1* functions in ferroptosis, but one hypothesis is that higher levels of *AL606489.1* may inhibit immunotherapy-dependent ferroptosis in male lung cancer patients. Due to its differential expression in male and female cancer patients, the lncRNA *AL606489.1* has been associated with a worsened prognosis specifically in males. This indicates potential interactions with unidentified proteins, suggesting a distinct functional role in male and female cancer cells. However, more studies are needed to understand the mechanisms of cancer sex-specific lncRNA functions and the possible cancer sex dimorphism in response to immunotherapy.

The lncRNA *SATB2-AS1* (SATB2 Antisense RNA 1) is also more abundant in male lung cancer patients compared to female patients, but overall survival does not differ between males and females (Liang et al. 2022). *SATB2-AS1* contributes to the composition of the tumor immune cell microenvironment, and *SATB2-AS1* expression is negatively correlated with the infiltration of immune cells in colorectal cancer (Xu et al. 2019). It has been found that male cancer patients have a lower immune cell infiltration than female cancer patients (Fan et al. 2021; Klein and Flanagan 2016; Laskar et al. 2021). Male colorectal cancer patients have a high level of *SATB2-AS1* expression, which may contribute to the low immune cell infiltration observed. However, experimental studies are needed to validate a direct connection between the lncRNA *SATB2-AS1* and the effects on the tumor immune cell microenvironment. The immune system plays a crucial role in cancer progression, and the sex dimorphism that underlying that hallmark calls for a closer look at the immune sex difference driven by lncRNAs. Cancer immunotherapy could be improved by unraveling how lncRNAs function in sexual dimorphic immune responses. Identifying sex-specific immune-related lncRNAs could help prognosis and adapt treatment accordingly (Isaev et al. 2021).

## Identification of novel lncRNAs in cancer sex dimorphism

High-throughput sequencing has revealed that the majority of cancer-related gene variations are in the non-coding regions of the human genome with only a small amount found in protein-coding regions. With documented molecular functions and observed high degree of expression specificity, some lncRNAs have been proposed as promising biomarkers and therapeutic targets in cancer. However, the utilization of lncRNAs as reliable biomarkers remains a challenging endeavor. Some lncRNAs have already found application as diagnostic biomarkers; one notable example is *PCA3*, which is employed in the PROGENSA *PCA3* urine test for prostate cancer diagnosis ﻿(Li et al. 2023b). Another promising diagnostic biomarker is *MALAT-1*, known for its high expression in the plasma of patients with prostate cancer (Wang et al. 2017). In various other types of cancer, several lncRNAs are currently under investigation as potential prognostic biomarkers, such as *XLOC_014172* and *LOC149086* in hepatocellular carcinoma (Qi et al. 2016). Furthermore, in the realm of therapeutic biomarkers, pre-clinical studies have demonstrated the efficacy of targeting *MALAT-1* using RNA interference in prostate cancer, yielding successful results in xenograft models (Wang et al. 2017). Nevertheless, the discovery of cancer sex-specific lncRNAs remains imperative for the development of reliable diagnostic, prognostic, or therapeutic biomarkers. Two main strategies exist for identifying cancer biomarkers: the candidate gene approach and the genetic screen approach. Each of these approaches can identify any cancer biomarker, but integrating specific features can allow them to identify sex-specific lncRNAs.

### Candidate gene approach

With the availability of sequencing data, it is reasonable to identify candidate genes with a higher degree of statistical significance. The Cancer Genome Atlas (TCGA) contains over 85,000 sequencing data along with clinical information (The Cancer Genome Atlas Research Network 2011). It has already been used to identify coding genes associated with sex-cancer dimorphisms (Li et al. 2020). Identifying the lncRNA differentially expressed between males and females across different cancers is possible thanks to the growing number of cancer lncRNA databases. A user-friendly database such as The Atlas of NcRNA In Cancer (TANRIC), for example, explores the expression of lncRNAs in cancer and compares them with clinical information such as sex (Li et al. 2015). However, one challenge with lncRNAs is that they are not adequately annotated. The FAMTOM6 (Functional ANnoTation Of the Mammalian genome) project aims to annotate lncRNAs (Ramilowski et al. 2020). Incorporating the FANTOM6 annotation database with TCGA data enables one to identify additional lncRNAs associated with cancer-related sex dimorphism. A combination of the TANRIC, FANTOM6, and TCGA datasets can also be used to identify additional lncRNAs, providing valuable insights. For example, machine learning can be used to identify prognostic lncRNAs in TCGA and FANTOM databases (Isaev et al. 2021). In this pan-cancer study, *HOXA10-AS* (HOXA10 Antisense RNA) was identified as an lncRNA that could be used as a prognostic marker in low-grade gliomas. The loss-of-function experiments in patient cell gliomas showed that *HOXA10-AS* reduces cell proliferation. Data from RNA-sequencing indicated that the *HOXA10-AS* is involved in multiple hallmarks of cancer, including deregulation of the cell cycle pathway and the organization of the extracellular matrix (Isaev et al. 2021). This is an example of a methodology that can be used to identify cancer-specific lncRNAs. In addition to *HOXA10-AS*, the study identified 165 prognostic lncRNAs across a variety of cancer types. As machine-learning technology becomes more advanced, it is possible to query lncRNA databases to determine which lncRNAs are differentially expressed between males and females. For further validation, lncRNAs with sexual dimorphism regarding patient survival could be selected. Validation in vitro includes gain- and loss-of-function experiments using cancer cell lines, and in vivo validation involves the study of tumor formation using a mouse model.

### Genetic screen approach

CRISPR-gene editing is a reliable technique for performing large genetic screens, whether it is in vivo (using mouse models) or in vitro (using established cancer cell lines). A non-coding targeting screen can be performed on lncRNA–CRISPR libraries (Liu et al. 2018). It is also possible to use custom-made libraries to screen for specific lncRNA targets. CRISPR screens can be readily adapted to gain- or loss-of-function experiments (Joung et al. 2017; Liu et al. 2017; Prolo et al. 2019). However, one pitfall of CRISPR screens detecting lncRNA is that they are insensitive to reading frame alteration, leading to false-negative hits. A study has developed a library of sgRNA targeting lncRNA splicing sites to overcome this problem. There are more than 10,000 sgRNAs in this library, each of which targets either the splice donor (SD) site or the splice acceptor (SA) site of a given lncRNA. Using this strategy, specific intron retention (SD) or exon skipping (SA) can be induced, thereby disrupting the targeted lncRNA’s function (Liu et al. 2017). Large genomic screens can also be performed using Antisense Oligo Nucleotide (ASO) and RNA interference (RNAi) (Yip et al. 2022). To identify cancer sex-specific lncRNAs, a screen in female and male cancer cell lines must be performed. A variety of phenotypes can be asserted, such as survival, proliferation, invasion, and migration. A major advantage of the genetic screen approach is the ability to identify genes involved in tumor progression and compare their impact on female and male cells. Using cell lines has the disadvantage of missing all aspects of the tumor microenvironment (TME) that could impact sex-specific gene regulation. Therefore, clinical validation of the findings is essential.

## Summary

Personalized therapies make it increasingly important to take biological sex into account. This review shows that lncRNAs play a role in cancer progression with different outcomes based on the sex. The molecular mechanisms behind this dimorphism need to be understood. LncRNAs are promising therapeutic targets that can also be used as diagnostic tools. There are several pathways involving lncRNAs, including chromosome X inactivation, hormonal signaling, and immunity. However, it is important to note that these are all interconnected pathways. Indeed, it is well known that the immune system is dependent on the signaling of sex hormones’ signaling, and that some immune genes are expressed from the X chromosome, including *TLR8* (Toll-like receptor 8), *TLR7* (Toll-like receptor 7), and *IRAK1* (Interleukin-receptor-associated kinase 1). Moreover, it is difficult to determine how lifestyle influences cancer sex disparities and how environmental factors interact with sex-specific lncRNAs in affecting cancer. For the lncRNAs highlighted in this review, however, further investigation is required to establish a causal relationship with cancer sex dimorphism. The identification of the molecular mechanisms underlying cancer sex dimorphism may lead to the development of more targeted and effective cancer treatment options for males and females. In addition to its scientific impact, the characterization of sex dimorphism-related lncRNA could also have a positive impact on communities disproportionately at risk or afflicted by cancer. As a result of improving cancer treatment, we could potentially reduce cancer-related health disparities and improve health outcomes overall.

## References

[CR1] Abdel-Hafiz HA, Schafer JM, Chen X, Xiao T, Gauntner TD, Li Z, Theodorescu D (2023) Y chromosome loss in cancer drives growth by evasion of adaptive immunity. Nature 619(7970):624–631. 10.1038/s41586-023-06234-x 37344596 10.1038/s41586-023-06234-x PMC10975863

[CR2] Arun G, Diermeier S, Akerman M, Chang K-C, Wilkinson JE, Hearn S, Kim Y, MacLeod AR, Krainer AR, Norton L, Brogi E, Egeblad M, Spector DL (2016) Differentiation of mammary tumors and reduction in metastasis upon Malat1 lncRNA loss. Genes Dev 30(1):34–51. 10.1101/gad.270959.115 26701265 10.1101/gad.270959.115 PMC4701977

[CR3] Bhan A, Hussain I, Ansari KI, Bobzean SAM, Perrotti LI, Mandal SS (2014) Bisphenol-A and diethylstilbestrol exposure induces the expression of breast cancer associated long noncoding RNA HOTAIR in vitro and in vivo. J Steroid Biochem Mol Biol 141:160–170. 10.1016/j.jsbmb.2014.02.002 24533973 10.1016/j.jsbmb.2014.02.002 PMC4025971

[CR4] Bhan A, Soleimani M, Mandal SS (2017) Long non-coding RNA (LncRNA) and cancer: a new paradigm. Cancer Res 77(15):3965–3981. 10.1158/0008-5472.CAN-16-2634 28701486 10.1158/0008-5472.CAN-16-2634 PMC8330958

[CR5] Brockdorff N, Bowness JS, Wei G (2020) Progress toward understanding chromosome silencing by Xist RNA. Genes Dev 34(11–12):733–744. 10.1101/gad.337196.120 32482714 10.1101/gad.337196.120 PMC7263139

[CR6] Castro A, Pyke RM, Zhang X, Thompson WK, Day C-P, Alexandrov LB, Zanetti M, Carter H (2020) Strength of immune selection in tumors varies with sex and age. Nat Commun 11(1):4128. 10.1038/s41467-020-17981-0 32807809 10.1038/s41467-020-17981-0 PMC7431859

[CR7] Chaligné R, Popova T, Mendoza-Parra M-A, Saleem M-AM, Gentien D, Ban K, Piolot T, Leroy O, Mariani O, Gronemeyer H, Vincent-Salomon A, Stern M-H, Heard E (2015) The inactive X chromosome is epigenetically unstable and transcriptionally labile in breast cancer. Genome Res 25(4):488–503. 10.1101/gr.185926.114 25653311 10.1101/gr.185926.114 PMC4381521

[CR97] Chen Y, Sun Y, Rao Q, Hua X, Li L, Chang C (2015) Androgen receptor (AR) suppresses miRNA-145 to promote renal cell carcinoma (RCC) progression independent of VHL status. Oncotarget 6(31):31203–31215. 10.18632/oncotarget.4522 26304926 10.18632/oncotarget.4522 PMC4741598

[CR8] Chou K, Henderson J (2014) Endocrine system. In: Wexler P (ed) Encyclopedia of toxicology, 3rd edn. Academic Press, New York, pp 332–340. 10.1016/B978-0-12-386454-3.00377-8

[CR9] Clusan L, Ferrière F, Flouriot G, Pakdel F (2023) A basic review on estrogen receptor signaling pathways in breast cancer. Int J Mol Sci 24(7):6834. 10.3390/ijms24076834 37047814 10.3390/ijms24076834 PMC10095386

[CR10] Conforti F, Pala L, Pagan E, Bagnardi V, De Pas T, Queirolo P, Pennacchioli E, Catania C, Cocorocchio E, Ferrucci PF, Saponara M, Orsolini G, Zagami P, Nicoló E, De Marinis F, Tortora G, Bria E, Minucci S, Joffe H et al (2021) Sex-based dimorphism of anticancer immune response and molecular mechanisms of immune evasion. Clin Cancer Res 27(15):4311–4324. 10.1158/1078-0432.CCR-21-0136 34016641 10.1158/1078-0432.CCR-21-0136 PMC7611463

[CR11] Cuykendall TN, Rubin MA, Khurana E (2017) Non-coding genetic variation in cancer. Curr Opin Syst Biol 1:9–15. 10.1016/j.coisb.2016.12.017 30370373 10.1016/j.coisb.2016.12.017 PMC6203332

[CR12] Ding J, Yeh C-R, Sun Y, Lin C, Chou J, Ou Z, Chang C, Qi J, Yeh S (2018) Estrogen receptor β promotes renal cell carcinoma progression via regulating LncRNA HOTAIR-miR-138/200c/204/217 associated CeRNA network. Oncogene 37(37):5037–5053. 10.1038/s41388-018-0175-6 29789714 10.1038/s41388-018-0175-6

[CR13] Djebali S, Davis CA, Merkel A, Dobin A, Lassmann T, Mortazavi A, Tanzer A, Lagarde J, Lin W, Schlesinger F, Xue C, Marinov GK, Khatun J, Williams BA, Zaleski C, Rozowsky J, Röder M, Kokocinski F, Abdelhamid RF, Gingeras TR (2012) Landscape of transcription in human cells. Nature 489:7414. 10.1038/nature11233 10.1038/nature11233 PMC3684276 22955620

[CR14] Dorak MT, Karpuzoglu E (2012) Gender differences in cancer susceptibility: an inadequately addressed issue. Front Genet 3:268. 10.3389/fgene.2012.00268 23226157 10.3389/fgene.2012.00268 PMC3508426

[CR15] Fan T, Li C, He J (2021) Prognostic value of immune-related genes and comparative analysis of immune cell infiltration in lung adenocarcinoma: sex differences. Biol Sex Differ 12(1):64. 10.1186/s13293-021-00406-y 34863300 10.1186/s13293-021-00406-y PMC8642994

[CR16] Fang H, Disteche CM, Berletch JB (2019) X inactivation and escape: epigenetic and structural features. Front Cell Dev Biol 7:219. 10.3389/fcell.2019.00219 31632970 10.3389/fcell.2019.00219 PMC6779695

[CR17] Furlan G, Gutierrez Hernandez N, Huret C, Galupa R, van Bemmel JG, Romito A, Heard E, Morey C, Rougeulle C (2018) The Ftx noncoding locus controls X chromosome inactivation independently of its RNA products. Mol Cell 70(3):462-472.e8. 10.1016/j.molcel.2018.03.024 29706539 10.1016/j.molcel.2018.03.024

[CR18] Gong D, Chen M, Wang Y, Shi J, Hou Y (2022) Role of ferroptosis on tumor progression and immunotherapy. Cell Death Discov 8(1):1. 10.1038/s41420-022-01218-8 36289191 10.1038/s41420-022-01218-8 PMC9605952

[CR19] Guo Y, Qu Z, Li D, Bai F, Xing J, Ding Q, Zhou J, Yao L, Xu Q (2021) Identification of a prognostic ferroptosis-related lncRNA signature in the tumor microenvironment of lung adenocarcinoma. Cell Death Discov 7(1):1. 10.1038/s41420-021-00576-z 10.1038/s41420-021-00576-z PMC8313561 34312372

[CR20] Gupta RA, Shah N, Wang KC, Kim J, Horlings HM, Wong DJ, Tsai M-C, Hung T, Argani P, Rinn JL, Wang Y, Brzoska P, Kong B, Li R, West RB, van de Vijver MJ, Sukumar S, Chang HY (2010) Long non-coding RNA HOTAIR reprograms chromatin state to promote cancer metastasis. Nature 464(7291):1071–1076. 10.1038/nature08975 20393566 10.1038/nature08975 PMC3049919

[CR21] Hanahan D (2022) Hallmarks of cancer: new dimensions. Cancer Discov 12(1):31–46. 10.1158/2159-8290.CD-21-1059 35022204 10.1158/2159-8290.CD-21-1059

[CR22] Hanahan D, Weinberg RA (2011) Hallmarks of cancer: the next generation. Cell 144(5):646–674. 10.1016/j.cell.2011.02.013 21376230 10.1016/j.cell.2011.02.013

[CR23] Hartman RJG, Huisman SE, den Ruijter HM (2018) Sex differences in cardiovascular epigenetics—a systematic review. Biol Sex Differ 9(1):19. 10.1186/s13293-018-0180-z 29792221 10.1186/s13293-018-0180-z PMC5966883

[CR24] He D, Li L, Zhu G, Liang L, Guan Z, Chang L, Chen Y, Yeh S, Chang C (2014) ASC-J9 suppresses renal cell carcinoma progression by targeting an androgen receptor-dependent HIF2α/VEGF signaling pathway. Cancer Res 74(16):4420–4430. 10.1158/0008-5472.CAN-13-2681 24924778 10.1158/0008-5472.CAN-13-2681

[CR25] Henderson J (2005) Ernest starling and ‘hormones’: an historical commentary. J Endocrinol 184(1):5–10. 10.1677/joe.1.06000 15642778 10.1677/joe.1.06000

[CR26] Henley SJ, Ward EM, Scott S, Ma J, Anderson RN, Firth AU, Thomas CC, Islami F, Weir HK, Lewis DR, Sherman RL, Wu M, Benard VB, Richardson LC, Jemal A, Cronin K, Kohler BA (2020) Annual report to the nation on the status of cancer, part I: national cancer statistics. Cancer 126(10):2225–2249. 10.1002/cncr.32802 32162336 10.1002/cncr.32802 PMC7299151

[CR27] Hiam-Galvez KJ, Allen BM, Spitzer MH (2021) Systemic immunity in cancer. Nat Rev Cancer 21(6):6. 10.1038/s41568-021-00347-z 10.1038/s41568-021-00347-z PMC8034277 33837297

[CR28] Huang R, Zhu L, Zhang Y (2020) XIST lost induces ovarian cancer stem cells to acquire taxol resistance via a KMT2C-dependent way. Cancer Cell Int 20(1):436. 10.1186/s12935-020-01500-8 32943985 10.1186/s12935-020-01500-8 PMC7487955

[CR29] Isaev K, Jiang L, Wu S, Lee CA, Watters V, Fort V, Tsai R, Coutinho FJ, Hussein SMI, Zhang J, Wu J, Dirks PB, Schramek D, Reimand J (2021) Pan-cancer analysis of non-coding transcripts reveals the prognostic onco-lncRNA HOXA10-AS in gliomas. Cell Rep 37(3):109873. 10.1016/j.celrep.2021.109873 34686327 10.1016/j.celrep.2021.109873

[CR30] Islami F, Ward EM, Sung H, Cronin KA, Tangka FKL, Sherman RL, Zhao J, Anderson RN, Henley SJ, Yabroff KR, Jemal A, Benard VB (2021) Annual report to the nation on the status of cancer, part 1: national cancer statistics. JNCI J Natl Cancer Inst 113(12):1648–1669. 10.1093/jnci/djab131 34240195 10.1093/jnci/djab131 PMC8634503

[CR31] Issler O, van der Zee YY, Ramakrishnan A, Wang J, Tan C, Loh Y-HE, Purushothaman I, Walker DM, Lorsch ZS, Hamilton PJ, Peña CJ, Flaherty E, Hartley BJ, Torres-Berrío A, Parise EM, Kronman H, Duffy JE, Estill MS, Calipari ES et al (2020) Sex-specific role for the long non-coding RNA LINC00473 in depression. Neuron 106(6):912-926.e5. 10.1016/j.neuron.2020.03.023 32304628 10.1016/j.neuron.2020.03.023 PMC7305959

[CR32] Jacobson EC, Pandya-Jones A, Plath K (2022) A lifelong duty: how Xist maintains the inactive X chromosome. Curr Opin Genet Dev 75:101927. 10.1016/j.gde.2022.101927 35717799 10.1016/j.gde.2022.101927 PMC9472561

[CR33] Jiang S, Cheng S-J, Ren L-C, Wang Q, Kang Y-J, Ding Y, Hou M, Yang X-X, Lin Y, Liang N, Gao G (2019) An expanded landscape of human long noncoding RNA. Nucleic Acids Res 47(15):7842–7856. 10.1093/nar/gkz621 31350901 10.1093/nar/gkz621 PMC6735957

[CR34] Joung J, Konermann S, Gootenberg JS, Abudayyeh OO, Platt RJ, Brigham MD, Sanjana NE, Zhang F (2017) Genome-scale CRISPR-Cas9 knockout and transcriptional activation screening. Nat Protoc 12(4):828–863. 10.1038/nprot.2017.016 28333914 10.1038/nprot.2017.016 PMC5526071

[CR35] Jusic A, Salgado-Somoza A, Paes AB, Stefanizzi FM, Martínez-Alarcón N, Pinet F, Martelli F, Devaux Y, Robinson EL, Novella S (2020) Approaching sex differences in cardiovascular non-coding RNA research. Int J Mol Sci 21(14):4890. 10.3390/ijms21144890 32664454 10.3390/ijms21144890 PMC7402336

[CR36] Karner H, Webb C-H, Carmona S, Liu Y, Lin B, Erhard M, Chan D, Baldi P, Spitale RC, Sun S (2020) Functional conservation of LncRNA JPX despite sequence and structural divergence. J Mol Biol 432(2):283–300. 10.1016/j.jmb.2019.09.002 31518612 10.1016/j.jmb.2019.09.002

[CR37] Kirchgessner CU, Warren ST, Willard HF (1995) X inactivation of the FMR1 fragile X mental retardation gene. J Med Genet 32(12):925–929 8825916 10.1136/jmg.32.12.925 PMC1051769

[CR38] Klein SL, Flanagan KL (2016) Sex differences in immune responses. Nat Rev Immunol 16(10):626–638. 10.1038/nri.2016.90 27546235 10.1038/nri.2016.90

[CR39] Kopp F, Mendell JT (2018) Functional classification and experimental dissection of long noncoding RNAs. Cell 172(3):393–407. 10.1016/j.cell.2018.01.011 29373828 10.1016/j.cell.2018.01.011 PMC5978744

[CR40] Kumar S, Prajapati KS, Singh AK, Kushwaha PP, Shuaib M, Gupta S (2021) Long non-coding RNA regulating androgen receptor signaling in breast and prostate cancer. Cancer Lett 504:15–22. 10.1016/j.canlet.2020.11.039 33556545 10.1016/j.canlet.2020.11.039

[CR41] Laskar RS, Li P, Ecsedi S, Abedi-Ardekani B, Durand G, Robinot N, Hubert J-N, Janout V, Zaridze D, Mukeria A, Mates D, Holcatova I, Foretova L, Swiatkowska B, Dzamic Z, Milosavljevic S, Olaso R, Boland A, Deleuze J-F et al (2021) Sexual dimorphism in cancer: Insights from transcriptional signatures in kidney tissue and renal cell carcinoma. Hum Mol Genet 30(5):343–355. 10.1093/hmg/ddab031 33527138 10.1093/hmg/ddab031 PMC8098110

[CR99] Li J, Han L, Roebuck P, Diao L, Liu L, Yuan Y, Weinstein JN, Liang H (2015) TANRIC: an interactive open platform to explore the function of lncRNAs in cancer. Cancer Res 75(18):3728–3737. 10.1158/0008-5472.CAN-15-0273 26208906 10.1158/0008-5472.CAN-15-0273 PMC4573884

[CR42] Li M, Chai H-F, Peng F, Meng Y-T, Zhang L-Z, Zhang L, Zou H, Liang Q-L, Li M-M, Mao K-G, Sun D-X, Tong M-Y, Deng Z-Q, Hou Z-J, Zhao Y, Li J, Wang X-C, Lv S-S, Zhang Q-Q et al (2018) Estrogen receptor β upregulated by lncRNA-H19 to promote cancer stem-like properties in papillary thyroid carcinoma. Cell Death Dis 9(11):1120. 10.1038/s41419-018-1077-9 30389909 10.1038/s41419-018-1077-9 PMC6214949

[CR98] Li CH, Prokopec SD, Sun RX, Yousif F, Schmitz N, PCAWG Tumour Subtypes and Clinical Translation, Al-Shahrour F, Atwal G, Bailey PJ, Biankin AV, Boutros PC, Campbell PJ, Chang DK, Cooke SL, Deshpande V, Faltas BM, Faquin WC, Garraway L,Getz G et al (2020) Sex differences in oncogenic mutational processes. Nat Commun 11(1):4330. 10.1038/s41467-020-17359-2 10.1038/s41467-020-17359-2 PMC7455744 32859912

[CR43] Li J, Ming Z, Yang L, Wang T, Liu G, Ma Q (2022) Long noncoding RNA XIST: mechanisms for X chromosome inactivation, roles in sex-biased diseases, and therapeutic opportunities. Genes Dis 9(6):1478–1492. 10.1016/j.gendis.2022.04.007 36157489 10.1016/j.gendis.2022.04.007 PMC9485286

[CR44] Li J, Lan Z, Liao W, Horner JW, Xu X, Liu J, Yoshihama Y, Jiang S, Shim HS, Slotnik M, LaBella KA, Wu C-J, Dunner K, Hsu W-H, Lee R, Khanduri I, Terranova C, Akdemir K, Chakravarti D et al (2023a) Histone demethylase KDM5D upregulation drives sex differences in colon cancer. Nature 619(7970):632–639. 10.1038/s41586-023-06254-7 37344599 10.1038/s41586-023-06254-7 PMC10529424

[CR45] Li Y, Wei C, Huang C, Ling Q, Zhang L, Huang S, Liao N, Liang W, Cheng J, Wang F, Mo L, Mo Z, Li L (2023b) Long noncoding RNA as a potential diagnostic tool for prostate cancer: a systematic review and meta-analysis. Biomarkers 28(1):1–10. 10.1080/1354750X.2022.2142293 36323640 10.1080/1354750X.2022.2142293

[CR46] Liang J, Jin W, Xu H (2022) An efficient five-lncRNA signature for lung adenocarcinoma prognosis, with AL606489.1 showing sexual dimorphism. Front Genet 13:1052092. 10.3389/fgene.2022.1052092 36531243 10.3389/fgene.2022.1052092 PMC9748423

[CR47] Liu SJ, Horlbeck MA, Cho SW, Birk HS, Malatesta M, He D, Attenello FJ, Villalta JE, Cho MY, Chen Y, Mandegar MA, Olvera MP, Gilbert LA, Conklin BR, Chang HY, Weissman JS, Lim DA (2017) CRISPRi-based genome-scale identification of functional long noncoding RNA loci in human cells. Science 355(6320):aah7111. 10.1126/science.aah7111 27980086 10.1126/science.aah7111 PMC5394926

[CR48] Liu Y, Cao Z, Wang Y, Guo Y, Xu P, Yuan P, Liu Z, He Y, Wei W (2018) Genome-wide screening for functional long noncoding RNAs in human cells by Cas9 targeting of splice sites. Nat Biotechnol. 10.1038/nbt.4283 30395134 10.1038/nbt.4283

[CR49] Liu S, Lai W, Shi Y, Liu N, Ouyang L, Zhang Z, Chen L, Wang X, Qian B, Xiao D, Yan Q, Cao Y, Liu S, Tao Y (2020) Annotation and cluster analysis of long noncoding RNA linked to male sex and estrogen in cancers. Npj Precis Oncol 4(1):5. 10.1038/s41698-020-0110-5 32195358 10.1038/s41698-020-0110-5 PMC7054536

[CR50] Lu S, Shan N, Chen X, Peng F, Wang Y, Long H (2021) A novel immune-related long non-coding RNAs risk model for prognosis assessment of lung adenocarcinoma. Aging 13(23):25550–25563. 10.18632/aging.203772 34905504 10.18632/aging.203772 PMC8714149

[CR51] Lyon MF (1961) Gene action in the X-chromosome of the mouse (*Mus**musculus* L.). Nature 190:4773. 10.1038/190372a0 10.1038/190372a0 13764598

[CR52] Mattick JS, Makunin IV (2006) Non-coding RNA. Hum Mol Genet 15(suppl 1):R17–R29. 10.1093/hmg/ddl046 16651366 10.1093/hmg/ddl046

[CR53] Mattick JS, Amaral PP, Carninci P, Carpenter S, Chang HY, Chen L-L, Chen R, Dean C, Dinger ME, Fitzgerald KA, Gingeras TR, Guttman M, Hirose T, Huarte M, Johnson R, Kanduri C, Kapranov P, Lawrence JB, Lee JT et al (2023) Long non-coding RNAs: definitions, functions, challenges and recommendations. Nat Rev Mol Cell Biol. 10.1038/s41580-022-00566-8 36596869 10.1038/s41580-022-00566-8 PMC10213152

[CR54] Nair L, Chung H, Basu U (2020) Regulation of long non-coding RNAs and genome dynamics by the RNA surveillance machinery. Nat Rev Mol Cell Biol 21(3):123–136. 10.1038/s41580-019-0209-0 32020081 10.1038/s41580-019-0209-0 PMC7107043

[CR55] Patrat C, Ouimette J-F, Rougeulle C (2020) X chromosome inactivation in human development. Development 147(1):dev183095. 10.1242/dev.183095 31900287 10.1242/dev.183095

[CR56] Payer B, Lee JT (2008) X chromosome dosage compensation: how mammals keep the balance. Annu Rev Genet 42(1):1. 10.1146/annurev.genet.42.110807.091711 18729722 10.1146/annurev.genet.42.110807.091711

[CR57] Payer B, Lee JT, Namekawa SH (2011) X-inactivation and X-reactivation: epigenetic hallmarks of mammalian reproduction and pluripotent stem cells. Hum Genet 130(2):265–280. 10.1007/s00439-011-1024-7 21667284 10.1007/s00439-011-1024-7 PMC3744832

[CR58] Pellicoro A, Ramachandran P, Iredale JP, Fallowfield JA (2014) Liver fibrosis and repair: Immune regulation of wound healing in a solid organ. Nat Rev Immunol 14(3):181–194. 10.1038/nri3623 24566915 10.1038/nri3623

[CR59] Prolo LM, Li A, Owen SF, Parker JJ, Foshay K, Nitta RT, Morgens DW, Bolin S, Wilson CM, Vega LJCM, Luo EJ, Nwagbo G, Waziri A, Li G, Reimer RJ, Bassik MC, Grant GA (2019) Targeted genomic CRISPR-Cas9 screen identifies MAP4K4 as essential for glioblastoma invasion. Sci Rep 9(1):14020. 10.1038/s41598-019-50160-w 31570734 10.1038/s41598-019-50160-w PMC6768851

[CR60] Pyfrom S, Paneru B, Knox JJ, Cancro MP, Posso S, Buckner JH, Anguera MC (2021) The dynamic epigenetic regulation of the inactive X chromosome in healthy human B cells is dysregulated in lupus patients. Proc Natl Acad Sci USA 118(24):e2024624118. 10.1073/pnas.2024624118 34103397 10.1073/pnas.2024624118 PMC8214693

[CR61] Qi P, Zhou X, Du X (2016) Circulating long non-coding RNAs in cancer: current status and future perspectives. Mol Cancer 15(1):39. 10.1186/s12943-016-0524-4 27189224 10.1186/s12943-016-0524-4 PMC4869386

[CR62] Quinn JJ, Chang HY (2016) Unique features of long non-coding RNA biogenesis and function. Nat Rev Genet 17(1):1. 10.1038/nrg.2015.10 26666209 10.1038/nrg.2015.10

[CR63] Ramilowski JA, Yip CW, Agrawal S, Chang J-C, Ciani Y, Kulakovskiy IV, Mendez M, Ooi JLC, Ouyang JF, Parkinson N, Petri A, Roos L, Severin J, Yasuzawa K, Abugessaisa I, Akalin A, Antonov IV, Arner E, Bonetti A et al (2020) Functional annotation of human long noncoding RNAs via molecular phenotyping. Genome Res 30(7):1060–1072. 10.1101/gr.254219.119 32718982 10.1101/gr.254219.119 PMC7397864

[CR64] Reinius B, Shi C, Hengshuo L, Sandhu KS, Radomska KJ, Rosen GD, Lu L, Kullander K, Williams RW, Jazin E (2010) Female-biased expression of long non-coding RNAs in domains that escape X-inactivation in mouse. BMC Genom 11(1):614. 10.1186/1471-2164-11-614 10.1186/1471-2164-11-614 PMC3091755 21047393

[CR65] Richart L, Picod-Chedotel M-L, Wassef M, Macario M, Aflaki S, Salvador MA, Héry T, Dauphin A, Wicinski J, Chevrier V, Pastor S, Guittard G, Le Cam S, Kamhawi H, Castellano R, Guasch G, Charafe-Jauffret E, Heard E, Margueron R, Ginestier C (2022) XIST loss impairs mammary stem cell differentiation and increases tumorigenicity through mediator hyperactivation. Cell 185(12):2164-2183.e25. 10.1016/j.cell.2022.04.034 35597241 10.1016/j.cell.2022.04.034

[CR66] Rinn JL, Chang HY (2012) Genome regulation by long noncoding RNAs. Annu Rev Biochem 81(1):145–166. 10.1146/annurev-biochem-051410-092902 22663078 10.1146/annurev-biochem-051410-092902 PMC3858397

[CR67] Roberts AL, Morea A, Amar A, Zito A, El-Sayed Moustafa JS, Tomlinson M, Bowyer RC, Zhang X, Christiansen C, Costeira R, Steves CJ, Mangino M, Bell JT, Wong CC, Vyse TJ, Small KS (2022) Age acquired skewed X chromosome inactivation is associated with adverse health outcomes in humans. Elife 11:e78263. 10.7554/eLife.78263 36412098 10.7554/eLife.78263 PMC9681199

[CR68] Rosspopoff O, Cazottes E, Huret C, Loda A, Collier AJ, Casanova M, Rugg-Gunn PJ, Heard E, Ouimette J-F, Rougeulle C (2023) Species-specific regulation of *XIST* by the *JPX/FTX* orthologs. Nucleic Acids Res 51(5):2177–2194. 10.1093/nar/gkad029 36727460 10.1093/nar/gkad029 PMC10018341

[CR69] Rubin JB, Lagas JS, Broestl L, Sponagel J, Rockwell N, Rhee G, Rosen SF, Chen S, Klein RS, Imoukhuede P, Luo J (2020) Sex differences in cancer mechanisms. Biol Sex Differ 11(1):17. 10.1186/s13293-020-00291-x 32295632 10.1186/s13293-020-00291-x PMC7161126

[CR70] Sadagopan A, Nasim IT, Li J, Achom M, Zhang C-Z, Viswanathan SR (2022) Somatic XIST activation and features of X chromosome inactivation in male human cancers. Cell Syst 13(11):932-944.e5. 10.1016/j.cels.2022.10.002 36356577 10.1016/j.cels.2022.10.002

[CR96] Sandovici I, Fernandez-Twinn DS, Hufnagel A, Constância M, Ozanne SE (2022) Sex differences in the intergenerational inheritance of metabolic traits. Nat Metabol 4(5):5. 10.1038/s42255-022-00570-4 10.1038/s42255-022-00570-4 35637347

[CR71] Schafer JM, Xiao T, Kwon H, Collier K, Chang Y, Abdel-Hafiz H, Bolyard C, Chung D, Yang Y, Sundi D, Ma Q, Theodorescu D, Li X, Li Z (2022) Sex-biased adaptive immune regulation in cancer development and therapy. iScience 25(8):104717. 10.1016/j.isci.2022.104717 35880048 10.1016/j.isci.2022.104717 PMC9307950

[CR72] Schmidt K, Carroll JS, Yee E, Thomas DD, Wert-Lamas L, Neier SC, Sheynkman G, Ritz J, Novina CD (2019) The lncRNA SLNCR recruits the androgen receptor to EGR1-bound genes in melanoma and inhibits expression of tumor suppressor p21. Cell Rep 27(8):2493-2507.e4. 10.1016/j.celrep.2019.04.101 31116991 10.1016/j.celrep.2019.04.101 PMC6668037

[CR73] Simchovitz-Gesher A, Soreq H (2020) Pharmaceutical implications of sex-related RNA divergence in psychiatric disorders. Trends Pharmacol Sci 41(11):840–850. 10.1016/j.tips.2020.09.003 33012545 10.1016/j.tips.2020.09.003

[CR74] Sohail SK, Sarfraz R, Imran M, Kamran M, Qamar S (2020) Estrogen and progesterone receptor expression in breast carcinoma and its association with clinicopathological variables among the Pakistani population. Cureus 12(8):e9751. 10.7759/cureus.9751 32944466 10.7759/cureus.9751 PMC7489794

[CR75] Sun S, Del Rosario BC, Szanto A, Ogawa Y, Jeon Y, Lee JT (2013) Jpx RNA activates Xist by evicting CTCF. Cell 153(7):1537–1551. 10.1016/j.cell.2013.05.028 23791181 10.1016/j.cell.2013.05.028 PMC3777401

[CR76] Syrett CM, Paneru B, Sandoval-Heglund D, Wang J, Banerjee S, Sindhava V, Behrens EM, Atchison M, Anguera MC (2019) Altered X-chromosome inactivation in T cells may promote sex-biased autoimmune diseases. JCI Insight 4(7):e126751. 10.1172/jci.insight.126751 30944248 10.1172/jci.insight.126751 PMC6483655

[CR77] Taheri M, Safarzadeh A, Hussen BM, Ghafouri-Fard S, Baniahmad A (2022) LncRNA/miRNA/mRNA network introduces novel biomarkers in prostate cancer. Cells 11(23):3776. 10.3390/cells11233776 36497036 10.3390/cells11233776 PMC9736264

[CR78] Tao S, He H, Chen Q (2015) Estradiol induces HOTAIR levels via GPER-mediated miR-148a inhibition in breast cancer. J Transl Med. 10.1186/s12967-015-0489-x 25928008 10.1186/s12967-015-0489-x PMC4421993

[CR95] The Cancer Genome Atlas Research Network (2011) Integrated genomic analyses of ovarian carcinoma. Nature 474(7353):609–615. 10.1038/nature10166 21720365 10.1038/nature10166 PMC3163504

[CR79] Tietze L, Kessler SM (2020) The good, the bad, the question-H19 in hepatocellular carcinoma. Cancers 12(5):1261. 10.3390/cancers12051261 32429417 10.3390/cancers12051261 PMC7281302

[CR80] Viggiano E, Ergoli M, Picillo E, Politano L (2016) Determining the role of skewed X-chromosome inactivation in developing muscle symptoms in carriers of Duchenne muscular dystrophy. Hum Genet 135(7):685–698. 10.1007/s00439-016-1666-6 27098336 10.1007/s00439-016-1666-6

[CR81] Wang R, Sun Y, Li L, Niu Y, Lin W, Lin C, Antonarakis ES, Luo J, Yeh S, Chang C (2017) Preclinical study using Malat1 small interfering RNA or androgen receptor splicing variant 7 degradation enhancer ASC-J9^®^ to suppress enzalutamide-resistant prostate cancer progression. Eur Urol 72(5):835–844. 10.1016/j.eururo.2017.04.005 28528814 10.1016/j.eururo.2017.04.005 PMC5802348

[CR82] Wutz A (2011) Gene silencing in X-chromosome inactivation: Advances in understanding facultative heterochromatin formation. Nat Rev Genet 12(8):8. 10.1038/nrg3035 10.1038/nrg3035 21765457

[CR83] Xing F, Liu Y, Wu S-Y, Wu K, Sharma S, Mo Y-Y, Feng J, Sanders S, Jin G, Singh R, Vidi P-A, Tyagi A, Chan MD, Ruiz J, Debinski W, Pasche BC, Lo H-W, Metheny-Barlow LJ, D’Agostino RB, Watabe K (2018) Loss of XIST in breast cancer activates MSN-c-Met and reprograms microglia via exosomal microRNA to promote brain metastasis. Cancer Res 78(15):4316–4330. 10.1158/0008-5472.CAN-18-1102 30026327 10.1158/0008-5472.CAN-18-1102 PMC6072593

[CR84] Xu M, Xu X, Pan B, Chen X, Lin K, Zeng K, Liu X, Xu T, Sun L, Qin J, He B, Pan Y, Sun H, Wang S (2019) LncRNA SATB2-AS1 inhibits tumor metastasis and affects the tumor immune cell microenvironment in colorectal cancer by regulating SATB2. Mol Cancer 18(1):135. 10.1186/s12943-019-1063-6 31492160 10.1186/s12943-019-1063-6 PMC6729021

[CR85] Xue X, Yang YA, Zhang A, Fong K-W, Kim J, Song B, Li S, Zhao JC, Yu J (2016) LncRNA HOTAIR enhances ER signaling and confers tamoxifen resistance in breast cancer. Oncogene 35(21):2746–2755. 10.1038/onc.2015.340 26364613 10.1038/onc.2015.340 PMC4791209

[CR86] Yager JD, Davidson NE (2006) Estrogen carcinogenesis in breast cancer. N Engl J Med 354(3):270–282. 10.1056/NEJMra050776 16421368 10.1056/NEJMra050776

[CR87] Yao R-W, Wang Y, Chen L-L (2019) Cellular functions of long noncoding RNAs. Nat Cell Biol 21(5):5. 10.1038/s41556-019-0311-8 10.1038/s41556-019-0311-8 31048766

[CR88] Yildirim E, Kirby JE, Brown DE, Mercier FE, Sadreyev RI, Scadden DT, Lee JT (2013) Xist RNA is a potent suppressor of hematologic cancer in mice. Cell 152:4. 10.1016/j.cell.2013.01.034 10.1016/j.cell.2013.01.034 PMC3875356 23415223

[CR89] Yip CW, Hon C-C, Yasuzawa K, Sivaraman DM, Ramilowski JA, Shibayama Y, Agrawal S, Prabhu AV, Parr C, Severin J, Lan YJ, Dostie J, Petri A, Nishiyori-Sueki H, Tagami M, Itoh M, López-Redondo F, Kouno T, Chang J-C et al (2022) Antisense-oligonucleotide-mediated perturbation of long non-coding RNA reveals functional features in stem cells and across cell types. Cell Rep 41(13):111893. 10.1016/j.celrep.2022.111893 36577377 10.1016/j.celrep.2022.111893

[CR90] Yu W, Ding J, He M, Chen Y, Wang R, Han Z, Xing EZ, Zhang C, Yeh S (2019) Estrogen receptor β promotes the vasculogenic mimicry (VM) and cell invasion via altering the lncRNA-MALAT1/miR-145-5p/NEDD9 signals in lung cancer. Oncogene 38(8):1225–1238. 10.1038/s41388-018-0463-1 30250297 10.1038/s41388-018-0463-1

[CR91] Yu B, Qi Y, Li R, Shi Q, Satpathy AT, Chang HY (2021) B cell-specific XIST complex enforces X-inactivation and restrains atypical B cells. Cell 184(7):1790-1803.e17. 10.1016/j.cell.2021.02.015 33735607 10.1016/j.cell.2021.02.015 PMC9196326

[CR92] Zhai W, Sun Y, Guo C, Hu G, Wang M, Zheng J, Lin W, Huang Q, Li G, Zheng J, Chang C (2017) LncRNA-SARCC suppresses renal cell carcinoma (RCC) progression via altering the androgen receptor(AR)/miRNA-143-3p signals. Cell Death Differ 24(9):1502–1517. 10.1038/cdd.2017.74 28644440 10.1038/cdd.2017.74 PMC5563985

[CR93] Zhang J, Fan D, Jian Z, Chen GG, Lai PBS (2015) Cancer specific long noncoding RNAs show differential expression patterns and competing endogenous RNA potential in hepatocellular carcinoma. PLoS One 10(10):e0141042. 10.1371/journal.pone.0141042 26492393 10.1371/journal.pone.0141042 PMC4619599

[CR94] Zhang Y, Pitchiaya S, Cieślik M, Niknafs YS, Tien JC-Y, Hosono Y, Iyer MK, Yazdani S, Subramaniam S, Shukla SK, Jiang X, Wang L, Liu T-Y, Uhl M, Gawronski AR, Qiao Y, Xiao L, Dhanasekaran SM, Juckette KM et al (2018) Analysis of the androgen receptor-regulated lncRNA landscape identifies a role for ARLNC1 in prostate cancer progression. Nat Genet 50(6):814–824. 10.1038/s41588-018-0120-1 29808028 10.1038/s41588-018-0120-1 PMC5980762

